# An inside out journey: biogenesis, ultrastructure and proteomic characterisation of the ectoparasitic flatworm *Sparicotyle chrysophrii* extracellular vesicles

**DOI:** 10.1186/s13071-024-06257-x

**Published:** 2024-04-03

**Authors:** Enrique Riera-Ferrer, Hynek Mazanec, Ivona Mladineo, Peter Konik, M. Carla Piazzon, Roman Kuchta, Oswaldo Palenzuela, Itziar Estensoro, Javier Sotillo, Ariadna Sitjà-Bobadilla

**Affiliations:** 1 Fish Pathology Group, Institute of Aquaculture Torre de La Sal, Consejo Superior de Investigaciones Científicas (IATS, CSIC), Ribera de Cabanes, 12595 Castellón, Spain; 2 Laboratory of Helminthology, Institute of Parasitology, Biology Centre, Czech Academy of Sciences, (BC CAS), České Budějovice, Czech Republic; 3 Laboratory of Functional Helminthology, Institute of Parasitology, Biology Centre Czech Academy of Sciences (BC CAS), České Budějovice, Czech Republic; 4 Faculty of Science, University of South Bohemia, Branišovská 1160/31, 370 05 České Budějovice, Czech Republic; 5 Parasitology Reference and Research Laboratory, National Centre for Microbiology, Instituto de Salud Carlos III, Majadahonda, Madrid, Spain

**Keywords:** Monogenea, Polyopisthocotyla, Exosomes, Ectosomes, Electron microscopy, Drug target candidates, Prophylactic target candidates, Peptidases

## Abstract

**Background:**

Helminth extracellular vesicles (EVs) are known to have a three-way communication function among parasitic helminths, their host and the host-associated microbiota. They are considered biological containers that may carry virulence factors, being therefore appealing as therapeutic and prophylactic target candidates. This study aims to describe and characterise EVs secreted by *Sparicotyle chrysophrii* (Polyopisthocotyla: Microcotylidae), a blood-feeding gill parasite of gilthead seabream (*Sparus aurata*), causing significant economic losses in Mediterranean aquaculture.

**Methods:**

To identify proteins involved in extracellular vesicle biogenesis, genomic datasets from *S. chrysophrii* were mined in silico using known protein sequences from *Clonorchis* spp., *Echinococcus* spp., *Fasciola* spp., *Fasciolopsis* spp., *Opisthorchis* spp., *Paragonimus* spp. and *Schistosoma* spp. The location and ultrastructure of EVs were visualised by transmission electron microscopy after fixing adult *S. chrysophrii* specimens by high-pressure freezing and freeze substitution. EVs were isolated and purified from adult *S. chrysophrii* (*n* = 200) using a newly developed ultracentrifugation-size-exclusion chromatography protocol for Polyopisthocotyla, and EVs were characterised via nanoparticle tracking analysis and tandem mass spectrometry.

**Results:**

Fifty-nine proteins involved in EV biogenesis were identified in *S. chrysophrii*, and EVs compatible with ectosomes were observed in the syncytial layer of the haptoral region lining the clamps. The isolated and purified nanoparticles had a mean size of 251.8 nm and yielded 1.71 × 10^8^ particles · mL^−1^. The protein composition analysis identified proteins related to peptide hydrolases, GTPases, EF-hand domain proteins, aerobic energy metabolism, anticoagulant/lipid-binding, haem detoxification, iron transport, EV biogenesis-related, vesicle-trafficking and other cytoskeletal-related proteins. Several identified proteins, such as leucyl and alanyl aminopeptidases, calpain, ferritin, dynein light chain, 14–3–3, heat shock protein 70, annexin, tubulin, glutathione *S*-transferase, superoxide dismutase, enolase and fructose-bisphosphate aldolase, have already been proposed as target candidates for therapeutic or prophylactic purposes.

**Conclusions:**

We have unambiguously demonstrated for the first time to our knowledge the secretion of EVs by an ectoparasitic flatworm, inferring their biogenesis machinery at a genomic and transcriptomic level, and by identifying their location and protein composition. The identification of multiple therapeutic targets among EVs' protein repertoire provides opportunities for target-based drug discovery and vaccine development for the first time in Polyopisthocotyla (sensu Monogenea), and in a fish-ectoparasite model.

**Graphical Abstract:**

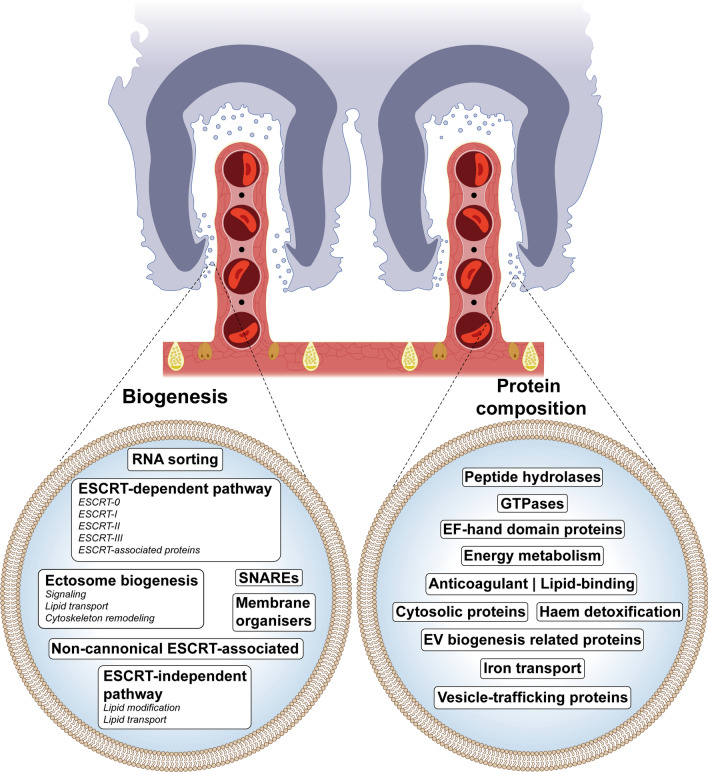

**Supplementary Information:**

The online version contains supplementary material available at 10.1186/s13071-024-06257-x.

## Background

Monogeneans, recently reclassified into two unrelated classes, Monopisthocotyla and Polyopisthocotyla [1, 2], are important aquatic, mainly ectoparasitic neodermates whose presence in fish farms pose a significant bottleneck for fish health, welfare, performance and economic revenue due to the artificial environment in which livestock is set [3].

Currently, the gill-infecting *Sparicotyle chrysophrii* (Van Beneden and Hesse, 1863) (Polyopisthocotyla: Microcotylidae) is among the most threatening pathogens in farmed gilthead seabream (*Sparus aurata*) across the Mediterranean Sea [4, 5]. The urge to find solutions for this parasitosis is clearly reflected in the scientific knowledge produced in the past decades. Epidemiological surveys [4] and distribution models have been established [6] and various anthelmintics have been tested [5, 7, 8].

Recently, a growing interest in sensu Monogenea secretome, composed of a soluble and a vesicular fraction, has emerged, leading to the in silico identification and characterisation of drug target candidates, such as peptidases and peptidase inhibitors considered as virulence factors, in several species [9–17]. In the last decade, the vesicular fraction of the secretome, which contains extracellular vesicles (EVs), has been extensively studied in human-associated and zoonotic flatworms and nematodes infecting mammals [18]. EVs have been assigned roles in host-parasite interactions, intraspecies communication and communication with the surrounding microbiota [18, 19].

EVs include exosomes and ectosomes, depending on their origin [20], and they follow different biogenesis pathways that ultimately determine the composition, cargo and function of EVs. Exosome formation following an endocytic pathway involves both the ESCRT (Endosomal Sorting Complex Required for Transport)-dependent and ESCRT-independent pathways and comprises intraluminal vesicles (ILVs) within multivesicular bodies (MVBs) [21–23]. The ESCRT-dependent pathway produces ILVs through tandem recruitment of members of the ESCRT machinery and associated proteins to the endosomal membrane, which subsequently control the process of membrane curvature as well as the cargo and final ILV scission. The ESCRT-independent pathway, on the other hand, requires proteins of the tetraspanin family for its regulation, sphingomyelinases to promote a spontaneous negative curvature of membranes and sphingosine kinase for the MVBs maturation and cargo loading [24]. Ectosomes, however, form as a result of direct outward budding and scission of the plasma membrane upon cytoskeletal rearrangement and Ca^2+^ influx as well as activation of scramblases, flippases and translocases [25].

The EV biogenesis machinery required for the different pathways is reported to be generally conserved in Neodermata. However, differences have been found between different classes. In particular, the machinery involved in the ESCRT-dependent pathway appears to be highly conserved in tapeworms (Cestoda), whereas it appears to be mostly incomplete in monogeneans (Popyopistocotyla), akin to Trematoda. Similarly, proteins involved in the non-canonical ESCRT-dependent pathway and ectosome formation are conserved in Neodermata. Conversely, the protein machinery required for the ESCRT-independent pathway appears to be the least conserved of all known pathways, regardless of the Neodermata class [26].

The heterogeneity of the helminth EV population is not only limited to its size but also to its protein composition, which differs depending on the site of formation. The role of helminth EVs in cell-to-cell communication involves the transfer of effector molecules including proteins, lipids, small RNAs (mRNA, miRNA and non-coding RNA species) and glycans [18]. The protein composition of EVs may include structural and biogenesis-related proteins as well as enzymatic proteins critical for parasite survival. Additionally, lipids within helminth vesicles not only contribute to the viability of EVs in the extracellular space but also may serve as bioactive immunomodulators. Furthermore, miRNAs are known to play immunomodulatory roles, while glycans are associated with EV internalisation [18]. Studies on helminth EVs have mainly focused on mammalian models. Notably, according to the latest research, EVs have been isolated only in the L3 stage of the nematode *Anisakis pegreffi* among marine fish parasites [27], adding another layer to the complexity of parasite-fish host-microbiome interactions.

In light of increasing resistance to known anthelmintics, EVs seem promising research targets due not only to their roles in essential parasite physiological processes but also to virulence factors contained in their cargo involved in the pathogenesis. In general, excretory-secretory products (ESPs), including EVs, are appealing targets for prophylactic, therapeutic and/or diagnostic purposes due to their extracellular localisation and theoretical presence in the host. Nevertheless, due to the co-evolution of parasitic helminths with their hosts, the mechanisms of action of essential biological functions such as parasite adhesion, invasion, nutrition or immune evasion may differ in each species. So far, EVs have been studied only in endoparasitic helminths (Cestoda, Trematoda and Nematoda), while no EVs have been studied in ectoparasitic flatworms. Though gill polyopisthocotylidans may have developed similar feeding and anchoring strategies, species-specific approaches are needed to decipher their unique pathogenic mechanisms so that effective therapies can be developed. In the current study, genomic and transcriptomic data of *S. chrysophrii* were generated for the identification of the protein machinery involved in different pathways of EV biogenesis and cargo sorting. The presence and tissular location of EVs in *S. chrysophrii* specimens was studied by transmission electron microscopy (TEM). Subsequently, EVs were isolated from adult *S. chrysophrii*, and their protein composition was characterised to identify therapeutic candidates for this parasite.

## Methods

### In silico scanning of proteins involved in extracellular vesicle biogenesis

**Fig. 1:**
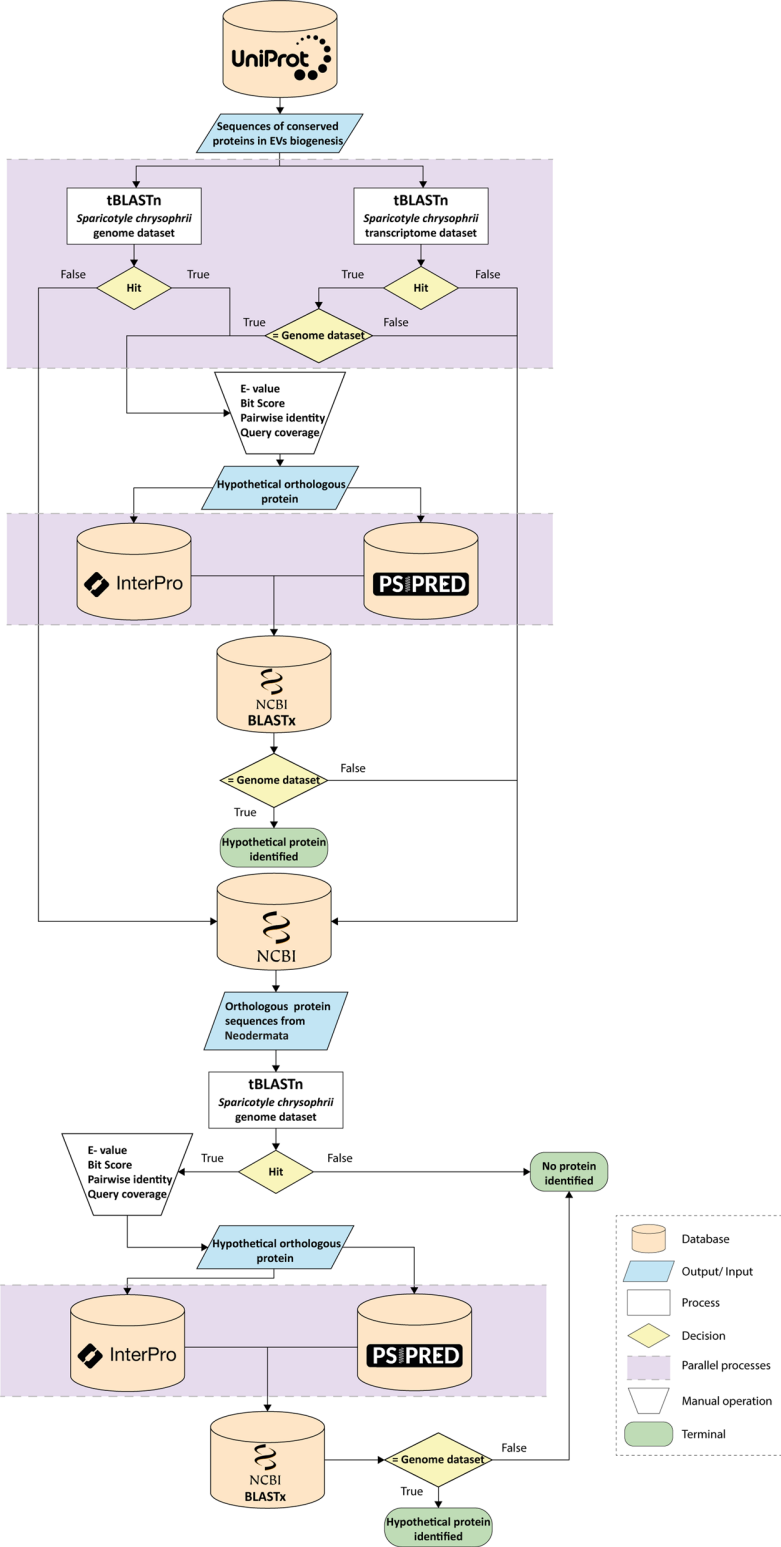
Workflow for in silico scanning of proteins involved in extracellular vesicle biogenesis. Protein sequences from *Homo sapiens* involved in EV biogenesis were collected from the UniProt database and used to interrogate *Sparicotyle chrysophrii* genome and transcriptome datasets, simultaneously using tBLASTn. Functional domains of *H. sapiens* and *S. chrysophrii* hypothetical orthologues were compared using InterPro, Pfam domains were retrieved, and PsiPred was used to compare their secondary structures. The chosen *S. chrysophrii* sequences were then used in a BLASTx search against the NCBI database. Orthologue protein sequences from Neodermata were used to interrogate *S. chrysophrii* draft genome by tBLASTn and the same methodology was applied to identify hypothetical proteins

A dataset of 82 sequences of known proteins involved in different EV biogenesis pathways in mammals (ESCRT-dependent pathway: 31 proteins; non-canonical ESCRT-associated pathway: 5 proteins; ESCRT-independent mechanisms: 21 proteins; proteins involved in ectosome formation: 9 proteins; EV membrane organisers: 9 proteins; and proteins involved in RNA sorting into EV: 7 proteins) [26] were retrieved from UniProt (www.uniprot.org) [28]. These sequences were used to interrogate the *S. chrysophrii* draft genome and transcriptome datasets by translated nucleotide basic alignment search tool (tBLASTn) [29] under the Geneious Prime v2023.1.2 software framework with default settings (BLOSUM62, gap cost: 11 1; limited to 20 hits). The best BLAST hits for each protein query were selected considering a combination of E-value, bit score, pairwise identity and query coverage values. The best hits were then aligned with the queries via multiple sequence comparison by log-expectation (MUSCLE), and functionally important domains were assessed and compared using the InterProScan plug-in. Additionally, the predicted secondary structure of the hits was assessed and compared to that of the human protein queries (PSIPRED 4.0; http://bioinf.cs.ucl.ac.uk/psipred) [30]. The hypothetical orthologues were then used as queries to the NCBI database (https://www.ncbi.nlm.nih.gov) by translated nucleotide sequence searched against protein sequences (BLASTx). When significant hits were not found in *S. chrysophrii* datasets, or when confident prediction of *S. chrysophrii* protein sequences from the aligned regions was not feasible, available neodermate homologues were retrieved from the NCBI database (https://www.ncbi.nlm.nih.gov). These homologues were sourced from *Clonorchis* spp., *Echinococcus* spp., *Fasciola* spp., *Fasciolopsis* spp., *Opisthorchis* spp., *Paragonimus* spp. and *Schistosoma* spp. because of the phylogenetic proximity among Trematoda, Cestoda and Polyopisthocotyla [1, 2] and considering their genomic quality based on individual genome assembly statistics. The same steps and criteria as described above were employed to interrogate *S. chrysophrii* datasets for these neodermate homologues. Finally, when different protein isoforms presented hit with the same *S. chrysophrii* contig, a single protein was considered (Additional file 1: Dataset S1). A representation of the entire data mining workflow is provided in Fig. 1.

### Parasite and fish maintenance

Gilthead seabream experimentally infected with *S. chrysophrii* were maintained in the Fish Pathology facilities at the Institute of Aquaculture Torre de la Sal under natural photoperiod and temperature conditions of the latitude (40°5’N; 0°10’E). The experimental infection was performed in a recirculating aquaculture system (RAS) where recipient *S. aurata* received water effluent from infected donor *S. aurata* tanks, and the infective pressure within the system and the experimental units was modulated using egg collectors [31]. Infected fish (*n* = 5) were euthanised by tricaine methanesulfonate (MS-222; Sigma, St. Louis, MO, USA) overexposure (0.1 g · L^−1^) and bled from the caudal vessels. Gill arches were dissected and adult *S. chrysophrii* specimens were gently detached from the gill filaments under a stereomicroscope using fine paintbrushes for subsequent in vitro maintenance. Additionally, a piece of one gill arch bearing adult parasite stages was fixed in 10% neutral buffered formalin, dehydrated in graded ethanol series for routine histological procedures, embedded in Technovit resin (Kulzer, Heraeus, Germany), 2 μm-sectioned, stained with toluidine blue and examined by light microscopy.

### Transmission electron microscopy (TEM)

Three adult *S. chrysophrii* specimens kept in vitro in complete Schnider’s *Drosophila* medium (non-supplemented with chicken serum) were collected and processed for high-pressure freezing (HPF) and freeze substitution as reported in Mladineo et al. [32]. After cold fixation, samples were washed in acetone 3 × for 15 min, infiltrated in mixtures of 25, 50 and 75% low viscosity Spurr resin (SPI Chem, West Chester, PA, USA) and anhydrous acetone for 1 h each and incubated overnight in 100% resin. The samples were left for polymerisation at 60 °C for 48 h in embedding moulds; afterwards, 1% toluidine blue-stained semi-thin sections (0.5 μm) were checked for orientation under the light microscope. Areas of interest were cut at 70 nm, mounted on Formvar-coated single-slot grids and contrasted for 30 min in ethanolic uranyl acetate and 20 min in lead citrate. Ultrathin sections were observed under a JEOL JEM-1400 microscope (JEOL, Akishima, Tokyo, Japan) operating at an accelerating voltage of 120 kV. The images were taken with a XAROSA 20-megapixel CMOS camera (EMSIS GmbH, Münster, Germany) and assembled and annotated in Inkscape 1.0 software (https://inkscape.org).

### Isolation and purification of extracellular vesicles

Two hundred adult *S. chrysophrii* specimens were kept in vitro for EV collection. Ten parasites per well were cultured in a 24 well-plate in 2.5 mL of 0.2 µm-filtered seawater (salinity 38 ppt) and kept at 18 °C. The medium from all wells was collected, pooled and replaced every 24 h for 3 days. Pooled samples from the three different sampling points (24 h, 48 h and 72 h) were processed separately. Initially, serial centrifugations were performed at 500, 2000 and 3500×*g* at 4°C for 30 min each, after which the supernatant containing ESPs was collected. The protein concentration in each ESP sample was quantified with the Pierce™ BCA protein assay kit (ThermoFisher Scientific, Waltham, MA, USA), and once the presence of proteins was confirmed, the samples were processed for a subsequent ultracentrifugation-size exclusion chromatography (UC-SEC) protocol.

ESPs were concentrated with an Amicon® Ultra-15 Centrifugal Filter Units 10 kDa (Amicon®, Miami, USA) to a final volume of 14 mL by centrifuging samples at 2500×*g* for 30 min at 12 °C. The resulting volume was centrifuged at 100,000×*g* for 8 h at 4 °C in a Beckman Optima XPN-90 Ultracentrifuge using a SW 40 Ti swinging-bucket rotor. The obtained pellets were resuspended in 500 µL of PBS and then processed by size-exclusion chromatography using qEVoriginal/70 nm Gen2 Columns (Izon Science, Christchurch, NZ). Finally, isolated EV samples were concentrated to a final volume of 250 µL by centrifuging samples at 3500×*g* for 1 h at 12 °C in Amicon® Ultra-4 Centrifugal Filter Units 10 kDa (Amicon®, Miami, FL, USA). In parallel, a 50 mL sample of the maintenance medium used and 0.2 µm-filtered PBS were processed similarly as a negative and quality control.

Isolated EV aliquots were diluted in PBS (1:5 *v/v*) and analysed via nanoparticle tracking analysis (NTA) using NanoSight (NS300; Malvern Panalytical, Malvern, UK) and software v3.4 to determine their nanoparticle concentration (particles · mL^−1^). The protein concentration from the resulting isolated EV samples was quantified with the Bradford protein assay (ThermoFisher Scientific, Waltham, USA) together with the nanoparticle concentration, their purity (particles · µg protein^−1^) was calculated according to Webber and Clayton [33].

### Extracellular vesicle protein cargo analysis and therapeutic target candidate exploration

For EV samples proteomic analysis, 20 µL of the sample was mixed 1:1 with 100 mM ammonium bicarbonate; Rapigest (Waters, Milford, MA, USA) surfactant was added at a final concentration of 0.1% (*v/v*) and incubated at 60 °C for 45 min. The mixture was cooled to room temperature, and proteomic grade trypsin (Sigma, St. Louis, USA) was added at a final concentration of 10 ng µL^−1^ and incubated at 37 °C. After 12 h, samples were acidified by addition of formic acid (Sigma, St. Louis, MO, USA) and peptides were isolated by StageTip procedure [34] to produce 30 µL of sample.

Liquid chromatography with tandem mass spectrometry (LC–MS/MS) was performed using an UltiMate 3000 UHPLC (ThermoFisher Scientific, Waltham, MA, USA) coupled online to a TimsTOF pro (Bruker, Billerica, MA, USA) mass spectrometer. Two µL of sample was trapped on a ThermoFisher trap column (0.3 × 5 mm, C18, 5 μm) for 1 min and then separated by reverse phase liquid chromatography on an Acclaim PepMap RSLC column (75 μm × 15 cm, C18, 2 μm, 100 Å; ThermoFisher Scientific, Waltham, MA, USA). The gradient ran for 30 min, during which the acetonitrile in 0.1% formic acid vs 0.1% formic acid ratio rose from 3 to 50%. Peptides were ionised using CaptiveSpray. Spectra were acquired in data-dependent PASEF (Parallel Accumulation/SErial Fragmentation) mode with an accuracy of 0.2 ppm for precursors and 0.5 ppm for fragments.

Raw data were processed using MaxQuant/Andromeda software [35, 36] and compared to a custom protein database constituted by a *S. chrysophrii* translation of our draft transcriptome and UniProt databases 9PLAT/37945 (Monogenea class specific proteins) and 9TELE/8174 (*Sparus* genus-specific proteins). Statistical analysis was performed with Perseus software v1.6.14.0 [37].

From the resulting statistical analysis, only the proteins with ≥ 2 unique peptides were considered for further analyses. Sequences of the selected proteins were retrieved from *S. chrysophrii* proteome and protein basic alignment search tool (BLASTp) was used against UniProt database with standard settings (BLOSUM62, gap cost: 11 1) to identify the proteins detected in the EV samples. The best BLAST hits for each protein query were selected considering a combination of E-value, bit score, pairwise identity and query coverage values, considering the E-value as the most determinant variable, limited to a value ≤ 1.00e−05. The hypothetical orthologues were further analysed for domain structure with InterPro (https://www.ebi.ac.uk/interpro) [38] and PfamScan (https://www.ebi.ac.uk/jdispatcher/pfa/pfamscan) [39], and for secondary structure with PSIPRED 4.0 (http://bioinf.cs.ucl.ac.uk/psipred) [30]. Furthermore, the identified proteins were used to perform BLASTp against the NCBI database (https://www.ncbi.nlm.nih.gov) with the aim to identify high-identity orthologue sequences from other haematophagous parasites, mainly in the Platyhelminthes, Nematoda and Arthropoda phyla.

Gene Ontology (GO) terms were obtained for each identified protein with Blast2GO v6.0.3 [40], and biological processes, molecular functions and cellular components were plotted using ReviGO v1.8.1 (http://revigo.irb.hr) [41], a web tool to visualise GO terms using semantic similarity-based scatterplots.

Finally, the proteins present in the EV samples were classified into non-enzymatic and enzymatic proteins and their corresponding subclasses with ECPred v1.1 [42]. Subsequently, those protein sequences classified within the peptide hydrolases/peptidases subclass underwent a BLASTp analysis with standard settings against the MEROPS database [43] to determine their peptidase nature.

## Results

### In silico scanning of proteins involved in extracellular vesicle biogenesis

Of the 82 selected and analysed known proteins involved in EV biogenesis pathways, a total of 59 proteins were identified in *S. chrysphrii* genome datasets. These proteins are represented in Table 1. Of the 31 known proteins that comprise the four ESCRT complexes (ESCRT-0, I, II and III) and ESCRT-accessory proteins of the ESCRT-dependent pathway, only one protein (VPS37) remained unidentified. Among the ESCRT-accessory proteins, although the PDCD6IP/ALIX protein appears to be present and its functional interaction domains were present, its homology with the protein orthologues of *Homo sapiens* and *Echinococcus granulosus* (Batsch, 1786) (Cestoda: Taeniidae) was low (E-value: 1.20E−01 and 1.41E−03, respectively; Additional file 1: Dataset S1). Within the ESCRT-dependent pathway, proteins involved in the non-canonical ESCRT-associated pathway were well conserved. In contrast, proteins involved in the ESCRT-independent pathway did not appear to be conserved in *S. chrysophrii*, as eight out of 14 proteins remained unidentified, among which S1PR1/3, SMPD1/3, SMPDL3a/b and PLA2 were identified.

**Table 1:** List of proteins involved in EV biogenesis present in *Sparicotyle chrysophrii*

Machinery	Gene name	Species	E-value genome	E-value transcriptome^1^
ESCRT-0	**HGS** ^a^	*Schistosoma mansoni*	6.38E−33	–
	**STAM** ^a^	*Schistosoma japonicum*	3.56E−19	–
	**STAMBP** ^a^	*Clonorchis sinensis*	2.92E−63	2.47E−57
ESCRT-I	**TSG101** ^a^	*Schistosoma japonicum*	2.70E−18	8.25E−22
	**VPS28** ^a^	*Schistosoma japonicum*	5.35E−35	1.09E−13
	**MVB12** ^a^	*Fasciola gigantica*	1.66E−40	–
ESCRT-II	**VPS SNF8** ^a^	*Paragonimus westermani*	2.22E−40	2.34E−66
	**VPS25** ^a^	*Schistosoma haematobium*	2.07E−74	–
	**VPS36** ^a^	*Homo sapiens*	7.08E−44	9.67E−48
ESCRT-III	**CHMP1a** ^a^	*Homo sapiens*	4.53E−41	2.05E−58
	**CHMP1b** ^a^	*Fasciolopsis buski*	2.40E−47	–
	**CHMP2a** ^a^	*Homo sapiens*	1.62E−24	–
	**CHMP2b** ^a^	*Homo sapiens*	1.10E−35	3.84E−41
	**CHMP3** ^a^	*Clonorchis sinensis*	2.04E−20	–
	**CHMP4** ^a^	*Homo sapiens*	3.17E−21	1.10E−32
	**CHMP5** ^a^	*Homo sapiens*	3.64E−46	–
	**CHMP6** ^a^	*Homo sapiens*	4.69E−10	1.48E−17
	**IST1** ^a^	*Homo sapiens*	2.97E−45	1.81E−57
ESCRT-associated proteins	**VPS4** ^a^	*Homo sapiens*	1.25E−45	0
	**VTA1** ^a^	*Paragonimus heterotremus*	8.08E−25	–
	ALIX ^a,^ ^b^	*Echinococcus granulosus*	1.41E−03	–
	**LITAF** ^a^	*Paragonimus heterotremus*	3.37E−11	–
Non-canonical ESCRT-associated	SDC1 ^b^	*Schistosoma mansoni*	1.70E−11	–
	**SDCBP** ^b^	*Homo sapiens*	1.80E−08	–
	**Ral-A** ^b^	*Homo sapiens*	6.66E−46	–
	**ARF6** ^b,^ ^d^	*Schistosoma japonicum*	8.52E−71	–
	**PLD2 **^**b**,^ ^d^	*Echinococcus granulosus*	8.28E−16	–
Lipid-modifying	**SMPD2**^c,^ ^d^	*Homo sapiens*	1.28E−04	8.86E−25
	**SMS2** ^c^	*Fasciola hepatica*	2.81E−105	–
	SPHK2 ^c^	*Schistosoma haematobium*	1.57E−11	–
	**LTA4H** ^c^	*Homo sapiens*	5.61E−140	1.74E−140
	DGK ^c^	*Homo sapiens*	3.63E−18	3.50E−58
Lipid transport	**ABCA **^c,^ ^d^	*Homo sapiens*	8.22E−117	3.30E−152
	**PLSCR** ^d^	*Schistosoma japonicum*	5.56E−12	–
	OSBP ^c^	*Schistosoma bovis*	2.81E−49	–
	**NPC1** ^c^	*Homo sapiens*	1.90E−15	2.07E−66
	**NPC2** ^c^	*Clonorchis sinensis*	4.55E−10	3.3E−28
Signalling	**ARF1** ^d^	*Homo sapiens*	1.13E−83	–
	**ROCK1** ^d^	*Fasciolopsis buski*	6.42E−65	–
	**ROCK2** ^d^	*Schistosoma japonicum*	1.24E−50	–
	**DIAPH3** ^d^	*Homo sapiens*	2.87E−62	1.87E−65
	**MYLK3** ^d^	*Paragonimus heterotremus*	0	2.99E−35
	**MAPK3** ^d^	*Fasciola hepatica*	2.06E−35	
Cytoskeleton remodelling	**CAPN**1 ^d^	*Homo sapiens*	1.22E−38	2.62E−70
	GELS ^d^	*Echinococcus multilocularis*	1.50E−10	3.74E−36
Membrane organisers	**CD63**	*Clonorchis sinensis*	2.09E−21	–
	**FLOT1**	*Homo sapiens*	2.13E−35	1.75E−115
	**FLOT2**	*Homo sapiens*	2.19E−28	1.54E−142
SNAREs	**STX1A**	*Echinococcus granulosus*	1.40E−42	–
	**STX5**	*Fasciola gigantica*	1.49E−17	–
	**YKT6**	*Paragonimus westermani*	9.34E−10	–
	**SYT1**	*Schistosoma japonicum*	3.20E−28	–
	**VAMP3**	*Homo sapiens*	4.34E−14	–
RNA sorting and transport	**hnRNPA2B1**	*Homo sapiens*	1.69E−52	6.73E−59
	**NSEP1**	*Homo sapiens*	1.83E−30	3.36E−38
	**hnRNPQ**	*Opisthorchis viverrini*	7.42E-90	–
	**Ago2**	*Homo sapiens*	1.48E−53	0
	**MVP**	*Schistosoma bovis*	1.06E−60	1.81E−116
Other vault complex proteins	PARP4	*Schistosoma mansoni*	4.92E−28	–

Lowercase superscript letters indicate the different EV biogenesis pathways. ^a^ESCRT-dependent pathway; ^b^non-canonical ESCRT-associated pathway; ^c^ESCRT-independent pathway; ^d^Ectosome formation pathways. 1: E-values from the transcriptome were solely obtained using *Homo sapiens* protein sequences. Bold lettering corresponds to protein sequences with ≥ 20% query coverage

According to the assessed proteins, the ectosome biogenesis pathway appeared to be preserved, with solely a single protein (ARRDC1) missing in *S. chrysophrii*. Soluble N-ethylmaleimide-sensitive factor-activating protein receptor (SNARE) proteins were well conserved in *S. chrysophrii*, VAMP7 being the only unidentified protein in the parasite. From all proteins analysed involved in cargo loading, such as those implicated in RNA sorting, i.e. hnRNPA2B1, NSEP1, hnRNPQ, Ago2, MVP and other vault-complex proteins (PARP4 and TEP1), the only unidentified protein in *S. chrysophrii* was TEP1.

### Light and transmission electron microscopy (TEM)

**Fig. 2:**
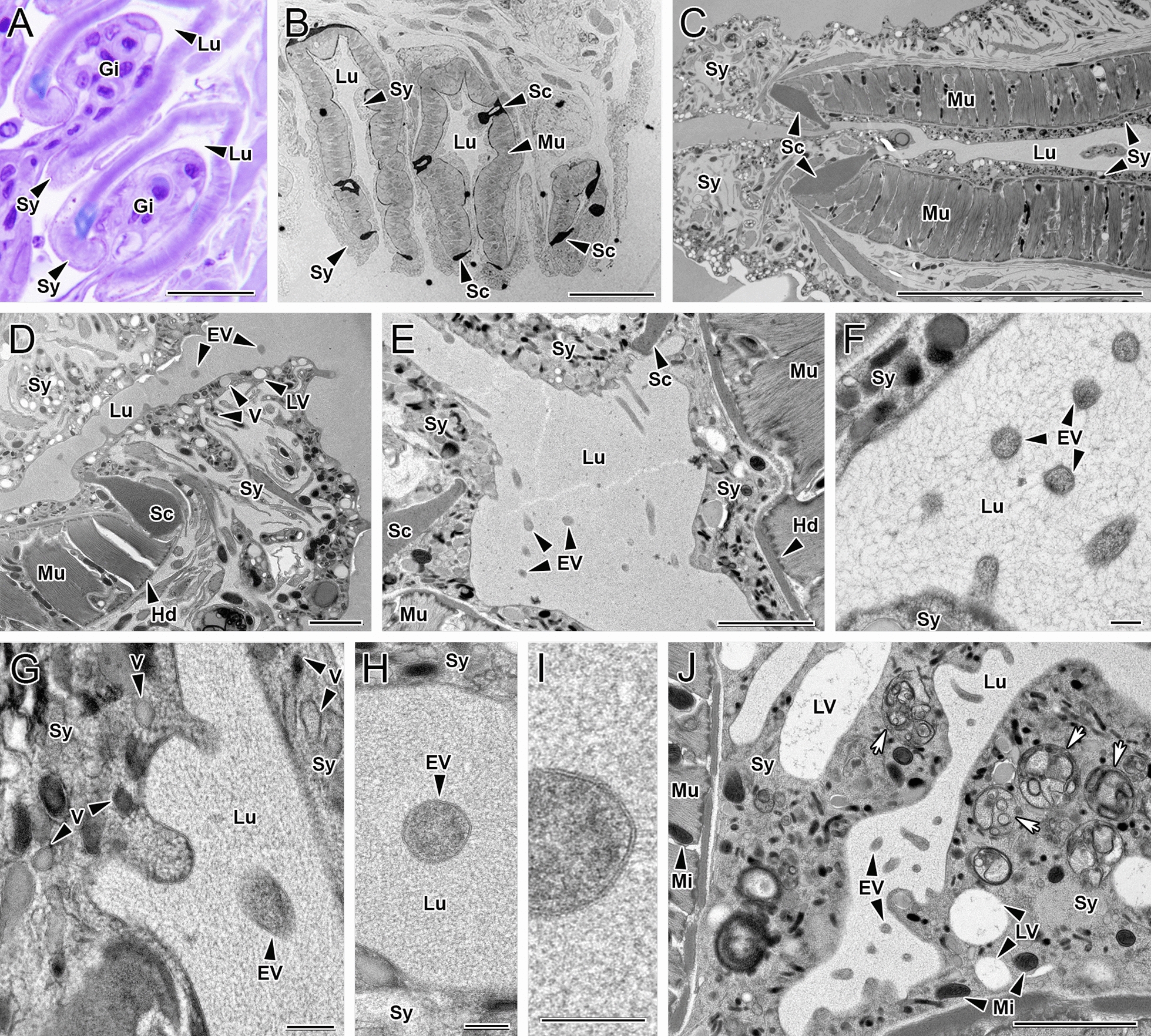
Microscopic images of the tegumental syncytial layer covering *Sparicotyle chrysophrii* clamps: **A** Light microscopy, **B**–**J** transmission electron microscopy. **A** Toluidine blue-stained section of gilthead sea bream (*Sparus aurata*) gill lamellae pinched by the parasite’s clamps. Note the syncytial layer covering the parasite’s clamps in close contact with the host lamellar tissue. **B** The three most distal clamps in the parasite’s opistohaptor. Note the syncytium covering the luminal and outer surface of the clamps. **C** Distal end of the clamp sclerites and associated myofilament bundles. Note the heterogeneity of the covering syncytium, which is a thin layer with flat surface at the clamp luminal side and thicker at the outer side and distal end, with an undulating surface. Higher magnification of the tegumental syncytium covering the clamp distal end at the tip (**D**) and luminal side (**E**). Note the presence of extracellular vesicles apparently emerging from the syncytial surface in the luminal part of the clamp and the abundance and diversity of vesicle types in the syncytium. **F** Detail of free extracellular vesicles in the clamp lumen, close to the clamp syncytial surface. **G** Detail of the undulating syncytial surface in the apparent process of secreting extracellular vesicles to the clamp lumen. **H**, **I** Two magnifications of a free extracellular vesicle in the clamp lumen, close to its syncytial surface. Note the bilayer enclosing coarse, moderately electrodense material. **J** Free extracellular vesicle in the clamp lumen in an area where the syncytium covering the clamp surface contains different vesicle types and multivesicular structures (arrows). Gi, gill filament; Hd, hemidesmosomes; Lu, clamp lumen; LV, large vacuoles; Mi, mitochondria; Mu, clamp myofilament bundles; EV, extracellular vesicles; Sc, sclerite; Sy, syncytial layer; V, syncytial vesicles. Scale bars: (A–C) = 20 µm, (D–E and J) = 2 µm, (F–I) = 200 nm

EVs with a diameter of approximately 200 nm consisting of a bilayer with coarse, moderately electrodense inner material are observed by TEM in the luminal space of clamps, either free or detaching from the tegumental syncytial layer lining the clamp surface (Fig. 2). The clamp ultrastructure consists of radially oriented bundles of myofilaments and mitochondria, extended among clamp sclerites. The syncytium, the outer layer of the tegument (neodermis), which covers the whole clamp sclerites and myofilaments, forms a thin layer (300 nm–2.5 µm) on the luminal clamp side. Abundant vesicles of different sizes and densities, granules, large vacuoles, mitochondria and multivesicular structures are observed in the cytoplasm of the EV-producing syncytium. The syncytium is also noticeable by light microscopy, especially at the clamp openings, in close contact with the gill lamellar tissues.

### Isolation and purification of extracellular vesicles

**Fig. 3:**
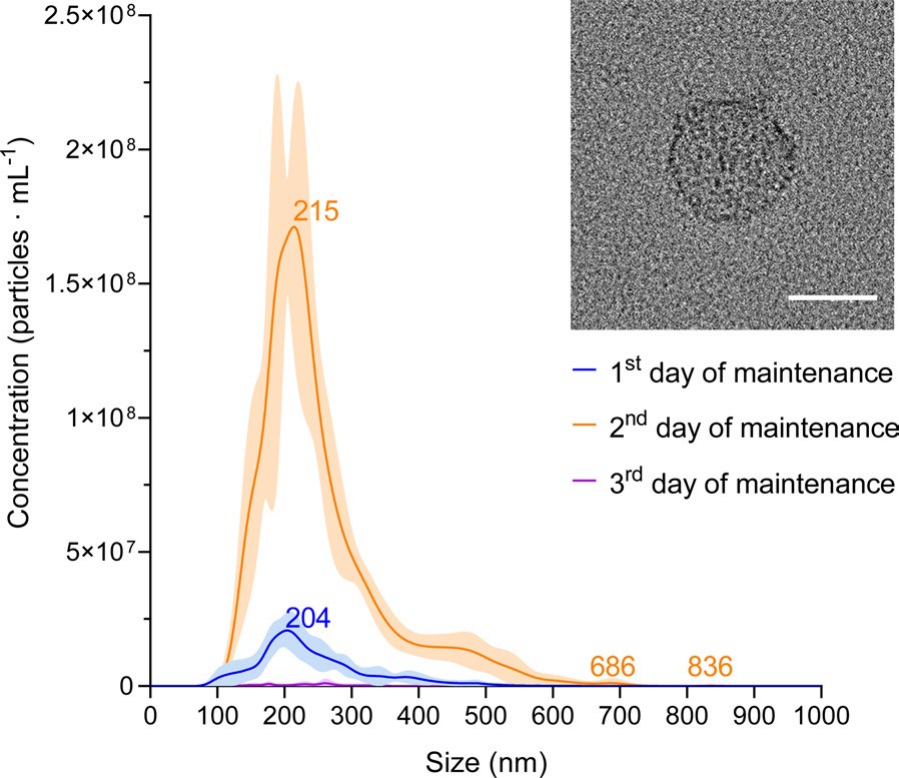
Nanoparticle concentration according to size of purified EVs from adult *Sparicotyle chrysophrii* collected after the 1st, 2nd and 3rd day of in vitro maintenance ± standard deviation. Insert shows an extracellular vesicle under transmission electron microscopy of the extracellular vesicle isolated samples. Scale bar = 200 nm

*Sparicotyle chrysophrii* specimens were kept for 3 days under in vitro conditions since parasites presented a mortality rate of 100% after the 3rd day without media supplementation. The NTA analyses indicated that the 2nd day of maintenance of *S. chrysophrii* was the most appropriate for EV collection, with the highest concentration and purity (Fig. 3; Table 2). The nanoparticle size ranged between 53.5 and 918.5 nm with a mean size of 251.8 ± 2.2 nm (± SD) and concentration peak at 215 nm (Fig. 3), yielding 1.71 × 10^8^ ± 4.55 × 10^7^ particles · mL^−1^ (mean ± SD; Additional file 2: Dataset S2). On the other hand, the concentration of particles · mL^−1^ in the maintenance medium without parasites was similar to filtered PBS, 1.53 × 10^7^ ± 5.23 × 10^6^ and 1.95 × 10^7^ ± 1.86 × 10^6^ particles · mL^−1^ (mean ± SD), respectively (Additional file 3: Fig. S1).

**Table 2:** Nanoparticle concentration, protein concentration and nanoparticle purity of purified EV samples collected at three sampling points during in vitro maintenance

In vitro maintenance (days)	Nanoparticle concentration (Mean particles · mL^−1^ ± SD)	Protein concentration (µg · mL^−1^)	Purity (particles · µg protein^−1^)
1	2.81 × 10^9^ ± 2.13 × 10^8^	8.602	3.27 × 10^8^
2	2.33 × 10^10^ ± 1.12 × 10^9^	33.840	6.89 × 10^8^
3	1.07 × 10^8^ ± 1.21 × 10^7^	1.343	7.97 × 10^7^

### Extracellular vesicle proteome analysis

A total of 124 proteins were successfully identified in EVs released by *S. chrysophrii*. From these, 18 proteins could putatively be involved in the different exosome and ectosome biogenesis pathways (including 8 small GTPases). Ten proteins were chaperone-related proteins; 11 were involved in energy metabolism processes; two were related to detoxification processes, 17 to hydrolases and one to iron transport (Additional file 4: Dataset S3).

**Fig. 4:**
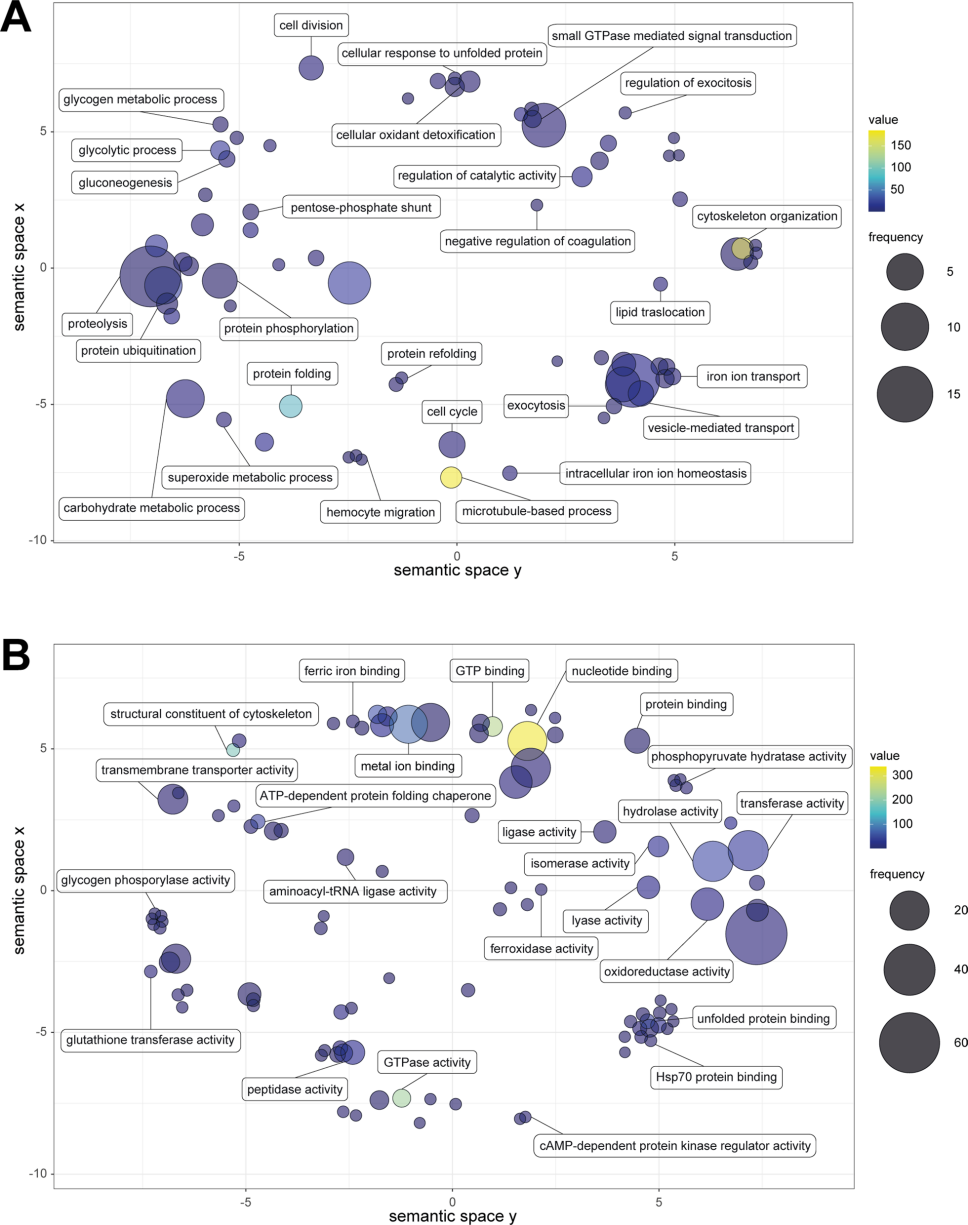
Gene ontology analysis of purified EV proteins from adult *Sparicotyle chrysophrii*. Semantically similar GO term scatterplots show the biological processes (**A**) and molecular function (**B**). The scatterplots show cluster representatives in a two-dimensional space where $x$ and $y$ coordinates are assigned to each GO term so that more semantically similar GO terms are set closer in the plot through a multidimensional scaling procedure. Circle size denotes the frequency of the GO term from the underlying database, and increasing heatmap score represents increasing node score from Blast2GO

After performing GO analysis, various metabolic processes were enriched, including the ones related to energy metabolism (glycolytic process, gluconeogenesis, pentose-phosphate pathway), proteolysis, protein ubiquitination, phosphorylation and folding processes. Additionally, cytoskeleton organisation and exocytosis processes, vesicle-mediated and iron transport, and negative regulation of coagulation were identified (Fig. 4A).

**Fig. 5:**
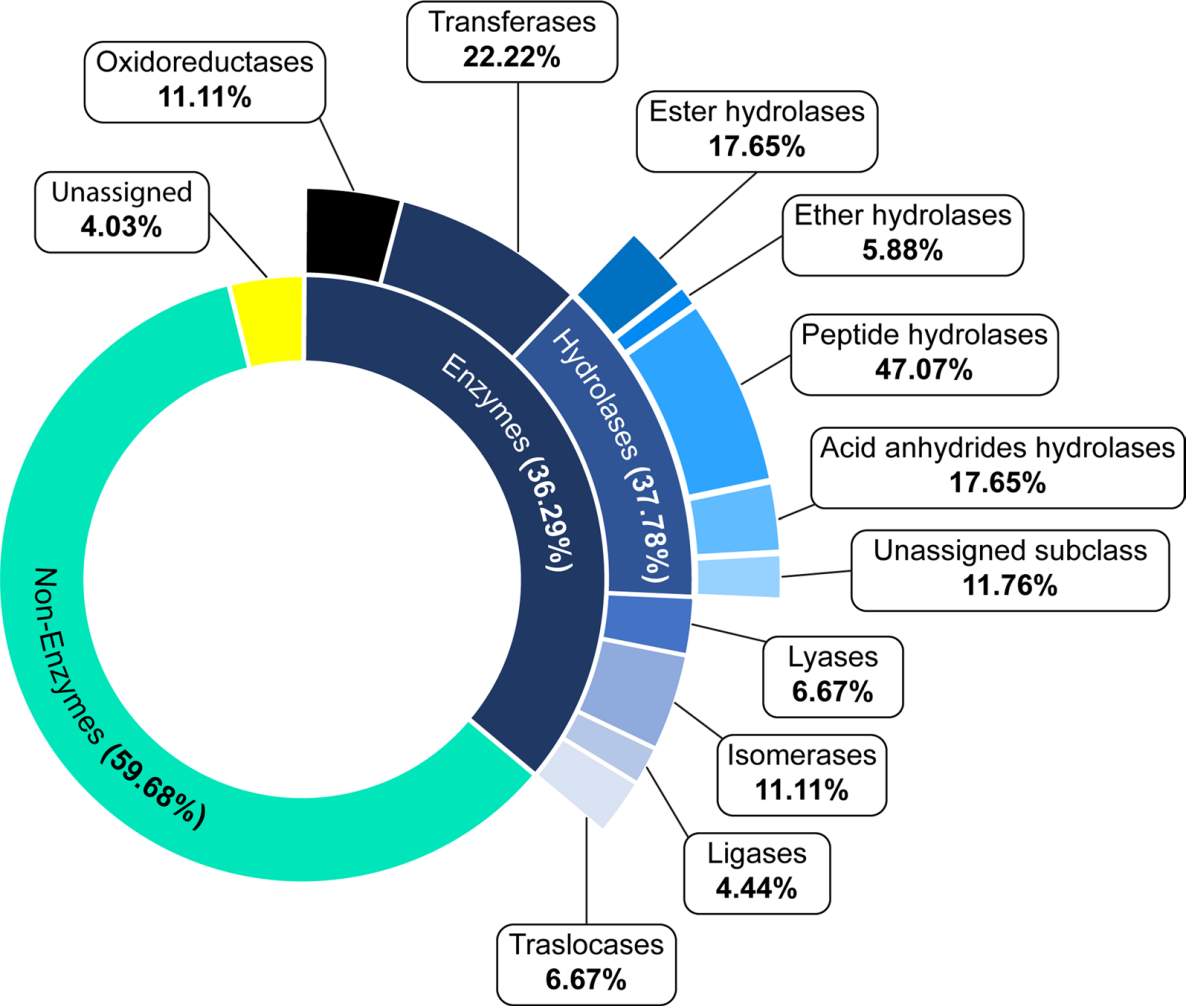
Enzymatic classification of the identified proteins in adult *Sparicotyle chrysophrii* EV samples. Represented percentages are calculated regarding each enzyme subclass

Regarding molecular functions, the analysis revealed glutathione transferase activity and catalytic activities such as oxidoreductase, transferase, hydrolase, lyase, isomerase and ligase activities, consistent with the results from the enzymatic classification analysis (Fig. 5). In addition, peptidase activity, metal ion and ferric iron binding, ferroxidase activity and protein and nucleotide binding, along with GTP and GTPase-related activities, were identified (Fig. 4B).

Moreover, the analysis indicated that cellular components related to the proteasome complex, the protein-containing complex and extracellular exosome proteins were represented (Additional file 5: Dataset S4).

Further in-depth analysis of the enzymatic nature of the proteins resulting from the proteome analysis identified 17 hydrolase sequences, attaining 37.78% of the total identified enzymes (Fig. 5), from which nine corresponded to peptidases: five proteasome subunits (threonine peptidases), three metallopeptidases and one cysteine peptidase were identified (Table 3).

**Table 3:** List of *Sparicotyle chrysophrii* peptidases present in EV samples, showing the peptidase clan and family according to MEROPS database, including their best hit

*Sparicotyle chrysophrii* protein sequence	Protein type	Peptidase		Species	E-value
		Clan	Family		
augustus_masked-contig_1681_pilon_pilon-processed-gene−0.0-mRNA-1	Leukotriene A-4 hydrolase^*^	MA	M1	*Schistosoma curassoni*	1.90e−175
maker-contig_20098_pilon_pilon-augustus-gene−0.16-mRNA-1	Leucyl aminopeptidase	MF	M17	*Fasciola* spp.	5.20e−157
maker-contig_22363_pilon_pilon-exonerate_est2genome-gene−0.0-mRNA-1	Proteasome subunit beta5i	PB	T1	*Schistosoma japonicum*	1.40e−120
maker-contig_24278_pilon_pilon-snap-gene−0.33-mRNA-1	Calpain	CA	C2	*Schistosoma margrebowiei*	1.80e−154
maker-contig_28754_pilon_pilon-snap-gene−0.18-mRNA-1	Proteasome alpha subunit G1	PB	T1	*Schistosoma mansoni*	3.40e−110
maker-contig_46585_pilon_pilon-augustus-gene−0.2-mRNA-1	Proteasome subunit alpha 1	PB	T1	*Clonorchis sinensis*	1.70e−130
maker-contig_4765_pilon_pilon-exonerate_est2genome-gene−0.1-mRNA-1	Proteasome alpha subunit G1	PB	T1	*Schistosoma mansoni*	2.90e−105
maker-contig_6883_pilon_pilon-snap-gene−0.23-mRNA-1	Cytosol alanyl aminopeptidase	MA	M1	*Echinococcus granulosus*	1.30e−138
maker-contig_7443_pilon_pilon-snap-gene−0.43-mRNA-1	Proteasome subunit alpha 6	PB	T1	*Schistosoma japonicum*	2.70e−62

^*^Indicates ether hydrolases with peptidase activity included in MEROPS database. **Peptidase clan**. MA: includes different families with aminopeptidase (M1, M16), carboxypeptidase (M2, M32), peptidyl-dipeptidase (M2), oligopeptidase (M3, M13) and endopeptidase activity (M4, M10, M12). MF: Presents solely proteins belonging to the M17 family, which have a predominant aminopeptidase activity. PB: Autocatalytic activation peptidase activity except for family T1. CA: Presents proteins with mostly endopeptidase activities; however, proteins belonging to C1 family present a predominant exopeptidase activity. **Peptidase family**. M1: Peptidase family mainly containing metallo-catalytic aminopeptidases dependent on a single zinc ion. M17: Peptidase family containing metallo-catalytic aminopeptidases with co-catalytic metal ions. T1: Peptidase family containing the peptidases components of proteasomes and related compound peptidases holding a threonine catalytic site. C2: Peptidase family containing endopeptidases termed “calpains” which hold a cysteine catalytic site

## Discussion

### Extracellular vesicle biogenesis

Most studies regarding EV biogenesis in neodermates have focused on the identification of the different proteins involved disregarding their biological functionality. Thus, neodermate EV biogenesis in general remains poorly understood and has not been addressed in Polyopisthocotyla (sensu Monogenea) fish parasites before.

Recently, the proteins required for EV biogenesis in helminths have been considered well conserved throughout different phyla. However, the degree of conservation of these proteins in Polyopisthocotyla, with *Protopolystoma xenopodis* (Price, 1943) (Polyopisthocotyla: Polystomatidae) as the only representative of such class, raised questions due to the lack of orthologues and the low conservation of their interacting regions [26].

Regarding EV biogenesis by the ESCRT-dependent pathway, ESCRT-0, ESCRT-II and ESCRT auxiliary proteins are almost fully conserved in Neodermata, whereas some proteins involved in ESCRT-I and ESCRT-III appear to be lacking in Trematoda (ESCRT-I: VPS28, VPS37 and MVB12; ESCRT-III: CHMP6 and IST1) and Polyopisthocotyla (ESCRT-I: TSG101, VPS37 and MVB12; ESCRT-III: CHMP2, CHMP3, CHMP5 and IST1) [26]. In the in silico analysis of *S. chrysophrii*, the only missing protein required for EV biogenesis related to the ESCRT-dependent pathway seems to be VPS37 (ESCRT-I), as no orthologue of any *H. sapiens* isoform (UniProt: VPS37a: Q8NEZ2; VPAS37b: Q9H9H4; VPS37c: A5D8V6; VPS37d: Q86XT2) nor the VPS37 protein (GenBank: RTG90235.1) of *Schistosoma bovis* (Sonsino, 1876) (Trematoda: Schistosomatidae) could be confidently identified. Notably, FYVE and VHS domains for HGS (ESCRT-0) were found and a single isoform orthologue was found for STAM (ESCRT-0), MVB12 (ESCRT-I), CHMP4 (ESCRT-III) and VPS4 (ESCRT-related component) in *S. chrysophrii*, apparently as opposed to the several isoforms of these proteins present in *H. sapiens* (STAM1/2, MVB12a/b, CHMP4a/b/c and VPS4a/b). From the analysed SNAREs, YKT6 was identified unlike in *P. xenopodis*, and VAMP7 remained the only unidentified protein in *S. chrysophrii*, in agreement with most Nematoda and Neodermata [26]. In view of these findings, we hypothesise that exosome production in *S. chrysophrii* may be driven primarily by the ESCRT-dependent pathway as described for adult *Fasciola hepatica* (Linnaeus, 1758) (Trematoda: Fasciolidae) [44]. However, VPS37 is key for the modulation of the ternary complex formation of ESCRT-I and PDCD6IP/ALIX [45] and yet it is missing in *S. chrysophrii* and in trematodes, or at least highly divergent from its *H. sapiens* homologues. Specific species adaptations cannot be ruled out, and specific EV biogenesis pathways may acquire relevant roles depending on the parasite’s life stage as observed in *Schistosoma japonicum* (Katsurada, 1904) (Trematoda: Schistosomatidae) [46]. In the context of the ESCRT-dependent pathway in mammals, a non-canonical ESCRT-associated route revolving around the programmed cell death 6-interacting protein (PDCD6IP/ALIX) has been identified in EV biogenesis. PDCD6IP/ALIX recruits ESCRT-III and facilitates the sorting and delivery of tetraspanins to exosomes. Additionally, PDCD6IP/ALIX can associate with transmembrane syndecan proteins via syntenin, promoting membrane budding steps of ILVs biogenesis [47–49]. In the current study, protein representatives of the non-canonical ESCRT-associated signalling pathway were identified, including syndecan, syntenin and a Bro1-domain containing protein, herein classified as PDCD6IP/ALIX. However, due to low homology (Table 1), further studies on *S. chrysophrii* PDCD6IP/ALIX are required. Tetraspanin CD63 and other membrane organiser proteins such as flotillin1/2 (FLOT1/2) appeared to be conserved in *S. chrysophrii*, in agreement with previous in silico studies [26].

Lipid-modifying proteins, involved in the ESCRT-independent pathway and ectosome formation (Table 1), seem to be the least conserved in Polyopisthocotyla and other helminths, probably because of the inability of helminth species to synthesise fatty acids de novo [50]. Thus, SMPD1/3, SMPDL3a/b, S1PR1/3 and PLA2 remained unidentified in our analysis and were consistent with the finding in *P. xenopodis*. However, three further aspects were noticed. Unlike in *P. xenopodis*, SMS2 and sphingosine kinase (SPHK) were identified. Interestingly, PLB-like 2 protein, apparently conserved Nematoda and Neodermata, including *P. xenopodis* [26], remained unidentified in *S. chrysophrii*. In addition, a single orthologue of the DGK isoform was identified in *S. chrysophrii* as opposed to the two isoforms present in *H. sapiens*.

The release of ectosomes is regulated by small Rab GTPases, including Rho-associated coiled coil-containing protein kinase and the GTP-binding protein ADP-ribosylation factor 6 (ARF6), which have been identified as positive regulators of vesicle budding in cancer cells [25, 51]. In the current study, Rho-associated protein kinase 1 and 2 (ROCK1/2) were identified together with ARF6 and ARF1 (Table 1); interestingly, a third ARF protein besides ARF6/1 was identified in the protein cargo from *S. chrysophrii* EVs (Additional file 4: Dataset S3), raising questions about the possible presence of other ARF isoforms and their role in polyopisthocotylidan EVs. Additionally, DIAPH3, previously reported in the neodermates *Echinococcus multilocularis* (Leuckart, 1863) (Cestoda: Taeniidae) and *Schistosoma* spp. [26], was identified in *S. chrysophrii*, adding Polyopisthocotyla as the third class within Neodermata where DIAPH3 has been identified. Other signalling proteins such as ARRDC1, apparently absent in Neodermata, also remained absent in *S. chrysophrii*, once again in agreement with previous findings [26].

Finally, regarding the proteins required for RNA sorting into exosomes, hnRNPQ was previously reported to be exclusive to Nematoda [26]. However, it was also identified in the trematode *Opisthorchis viverrini* (Poirier, 1886) (Trematoda: Opisthorchiidae; GenBank: OON14851.1), of which a single orthologue was found in *S. chrysophrii*.

### Extracellular vesicle isolation and transmission electron microscopy (TEM)

The present study showed that the 2nd day of in vitro maintenance of *S. chrysophrii* was the best time frame for EV collection with a nanoparticle concentration of 2.33 × 10^10^ ± 1.12 × 10^9^ particles · mL^−1^ (mean ± SD) and a sample purity of 6.89 × 10^8^ particles µg · protein^−1^. Previous studies proposed that a purity of 1 × 10^8^ particles µg · protein^−1^ represent high-quality vesicular preparations from *Schistosoma* spp. and other helminth parasites [52]. Since the purity of the analysed samples is in the same order of magnitude, given the target organism, we consider the EV samples to have a high purity.

So far, the presence of two different EV subpopulations according to their biogenesis pathways (exosomes and ectosomes) have been described in adult trematode *Fasciola* spp., *Schistosoma mansoni* (Sambon, 1907) (Trematoda: Schistosomatidae) and *O. viverrini* [44, 53–55].

In the current study, nanoparticles between 53.50 and 918.50 nm were isolated (Additional file 2: Dataset S2), and the EV protein composition of *S. chrysophrii* indicated the presence of proteins potentially involved in exosome and ectosome biogenesis pathways [20]. Moreover, TEM revealed EVs of ≈ 200 nm in diameter apparently budding off the syncytial lining covering *S. chrysophrii* clamps at the opisthaptor region, suggesting a possible ectosome biogenesis. However, the limited evidence precludes a conclusive determination of their exact biogenesis pathway.

The clamp covering syncytial cytoplasm containing different vesicle types, multivesicular structures and large vacuoles has been described in several polyopisthocotylidans, and vesicles in the process of exocytosis from the syncytium surface were observed in *Gotocotyla bivaginalis* (Ramalingam, 1961) (Polyopisthocotyla: Gotocotylidae) and *Chimaericola leptogaster* (Leuckart, 1830) (Polyopisthocotyla: Chimaericolidae) [56–58]. For the latter, differences in the syncytial surface and composition were also described depending on its luminal or external orientation in the clamp, where the syncytial thickenings at the clamp distal tips were considered clamp lips. However, the excretory/secretory importance of the secreted vesicles was not further discussed. Yet, exocytosis of syncytial material, such as secretory granules, vesicles and vacuoles, was also suggested as a mechanism of polyopisthocotylidan protection against immunological, ionic and osmotic damage caused by gill mucus secretion or water forces [58]. In *S. chrysophrii*, the occurrence of EVs in the tegument syncytium-lamellar membrane interphase could suggest a protective mechanism, but considering the tight host-parasite-microbiota contact, a mechanism of modulation on the fish host or host-associated microbiota via parasite’s ESPs should not be ruled out.

Konstanzová et al. [57] found glandular cells with vesicles at the sclerite base, apparently responsible for the secreted vesicles in the syncytium cytoplasm, in *Paradiplozoon homoion* (Bychowsky and Nagibina, 1959) (Polyopisthocotyla: Diplozoidae). These authors described three clearly differentiated vesicle types by their electron density and diameter and suggested that vesicles may have a storage function. In our case, a more heterogenous vesicle population from 50 to 400 nm in diameter was found in the cytoplasm of the tegument syncytium, but the question of whether these have a storage or secretory fate remains unsolved. Regarding ultrastructure, Konstanzová et al. [57] and Mergo [59] also described that clamps were covered by a very thin syncytial layer compared to the overall body surface in *P. homoion* and *Diplostamenides spinicirrus* (MacCallum, 1918) (formerly *Microcotyle spinicirrus*; Polyopisthocotyla: Microcotylidae), respectively. The reduction of the syncytial thickness in clamps is a trait shared among other polyopisthocotylidans and was attributed to an adaptation to increase the grasping ability to the host tissue in the limited space between gill lamellae [56–59]. In *S. chrysophrii*, no tegumental MVB containing ILVs nor exosomes on the outer body surface were identified, perhaps due to the way these parasites interact with their host, as opposed to endoparasitic helminths. However, the lack of secretion of EVs through tegument in other regions of the parasite’s body attributed to the sample preparation should not be ruled out.

In any case, the intimate contact between *S. chrysophrii* clamps and their hosts’ secondary lamellae pinpoints the opisthaptor as an optimal area for host recognition and continuous parasite-host-microbiota cross-talk, and haemorrhagic mechanical microlessions inflicted by the haptoral clamps in the gill epithelium [60] might facilitate host exposure to EVs. Future research might focus on the ability of *S. chrysophrii* EVs to internalise into host cells, especially since a cell-polarity regulator protein (LLGL scibble cell polarity complex component 2) has been identified in the current EV proteome analysis [61].

### Extracellular vesicle proteome analysis

In haematophagous parasites haemoglobin serves as a nutrient-rich source for obtaining iron and amino acids through the digestion of globins. For this purpose, haematophagous parasites employ a diverse set of peptidases. This process involves a multienzymatic network cascade, featuring clan CA (cathepsins B, C and L) and AA (cathepsin D) cysteine peptidases, alongside aminopeptidases [62, 63]. So far, they have been identified in Nematoda, Trematoda, Cestoda [64–66], Monopisthocotyla and Polyopisthocotyla [9, 11, 15, 16].

The liver fluke, *F. hepatica*, exhibits an extracellular digestion phase in its gut lumen [67], and a similar process is presumed in *Eudiplozoon nipponicum* (Goto, 1891) (Polyopisthocotyla: Diplozoidae) [10]. However, Riera-Ferrer et al. [68] suggested that *S. chrysophrii* induces an intravascular haemolysis in its host, prior to ingesting the resulting blood meal. Thus, ESPs and EV-originating peptidases and peptidase inhibitors might have a crucial role in this parasite’s feeding strategies.

Gene Ontology analyses from the purified EVs indicated biological processes related to blood (negative regulation of coagulation, iron ion transport, haemocyte migration and intracellular iron ion homeostasis; Fig. 4A), consistent with the negative haemostatic impact [68, 69] and catalytic process (regulation of catalytic activity and proteolysis) observed in infected *S. aurata*. Molecular functions revealed ferric and metal ion binding, ferroxidase activity, glutathione transferase and enzymatic activities related to oxidoreductase, transferase, hydrolase-peptidase, lyase, isomerase, ligase and translocase enzymes (Fig. 4B). Eight peptidases, including calpain, involved in signal transduction, cellular differentiation, cytoskeletal remodelling, vesicular trafficking and ectosome biogenesis [70, 71], two metallo- catalytic-type aminopeptidases, namely leucyl aminopeptidase and alanyl aminopeptidase, thought to be involved in the final steps of haemoglobin catabolism in *Plasmodium falciparum* (Welch, 1897) (Aconoidasida: Plasmodiidae) [72, 73], and the aminopeptidase leukotriene A4 hydrolase, related to the ESCRT-independent EV biogenesis pathway and suggested to have an extracellular peptidase role [74, 75], were identified in *S. chrysophrii* EVs (Table 3). Despite it is tempting to suggest an intravascular haemolytic event taking place, given the current findings and previous studies, further mechanistic, functionality, protein characterisation and EV internalisation studies are required. Notably, threonine proteasome-related peptidases (clan PB, family T1; Table 3) are the most represented among the identified peptidases, aligning with the observations in *E. nipponicum* secretome [16] and *F. hepatica* [44], implying that isolated EVs present a substantial role in protein turnover.

Free haem groups resulting from haemoglobin digestion elicit high toxicity and oxidative stress. To ensure their viability, hematophagous parasites require a haem-detoxification route [76]. Glutathione *S*-transferase, identified in *S. chrysophrii* EVs (Additional file 4: Dataset S3; Fig. 4B), is known for engaging in haem catabolic processing [77, 78]. Such results are consistent with observations in Trematoda [44, 52, 79–81] and in *E. nipponicum* [16], suggesting a significance in the haem detoxification pathway within Polyopisthocotyla.

Along the same line, haemoglobin remains the primary source of iron acquisition for parasitic blood-feeders, critical for multiple biological processes [82]. However, precise iron homeostasis is crucial, as both deficiency and excess pose survival challenges [83, 84]. Ferritins, iron storage proteins, counteract the toxic free iron ions by binding to them [85]. In isolated *S. chrysophrii* EV samples, a single ferritin (EC 1.16.3.1) was identified (Additional file 4: Dataset S3). GO analyses inferred iron ion transport, intracellular iron ion homeostasis (Fig. 4A) and ferric ion binding, metal ion binding and ferroxidase activities related to molecular function (Fig. 4B). The origin of free iron is unknown; although it might result from haem catabolic detoxification by glutathione *S*-transferase, solid evidence is lacking. Interestingly, ferritins are among the most transcribed genes in *E. nipponicum* [16], and given their presence in EVs of several trematode species [52, 80, 86], it is suggested that these play a significant role in Trematoda.

Platelet-activating factor (PAF; 1-*O*-alkyl-2-acetyl-*sn*-glycero-3-phosphocholine) is a key inflammatory mediator in the mammalian immune system, promoting platelet aggregation and triggering various immune responses and cytokine synthesis [87, 88]. In this study, we identified platelet-activating factor acetyl hydrolase (PAF-AH; EC 3.1.1.47), which by removing the *sn*-2 acetyl group from PAF silences its biological activity [89]. Thus, PAF-AH might ultimately inhibit the host’s coagulation cascade and extravasation of granulocytes. GO analysis inferred a negative regulation of the coagulation (Fig. 4A), overall aligning with the impaired haemostasis and immune system in *S. chrysophrii*-infected *S. aurata* [68]. Nevertheless, a significant increase in eosinophilic granular cells, the functional equivalent of mammalian neutrophils in *S. aurata* [90], was determined from 28 to 50 days post-parasite exposure [31]. In this context, the previously mentioned identification of a leukotriene A4 hydrolase suggests that *S. chrysophrii* might also secrete eicosanoids as proinflammatory mediators, as described for *S. japonicum* [91, 92]. Furthermore, human PAF-AH has been described to bind to very low density lipoproteins (VLDL), intermediate density lipoproteins (IDL), low density lipoproteins (LDL) and high density lipoproteins (HDL) [93]. This is in line with the impact on the lipid transport and metabolism described in *S. chrysophrii*-infected *S. aurata*, which presented plasma apolipoprotein impairment and hypocholesterolaemia [68]. The role of PAF-AH in *Nippostrongylus brasilensis* (Yokogawa, 1920) (Nematoda: Heligmonellidae) was inconclusive [94], contrasting with *Leishmania major* (Yakimoff and Schokhor, 1914) (Kinetoplastea: Trypanosomatidae) where it acts as a virulence factor [95]. Altogether, these findings prompt further studies to explore the potential pathogenic roles of PAF-AH in *S. chrysophrii*.

The understanding of the energy metabolism in Polyopisthocotyla remains elusive. Previous studies on *S. chrysophrii*, *E. nipponicum* and *Diclidophora merlangi* (Kuhn, 1829) (Polyopisthocotyla: Diclidophoridae) suggested an oxygen requirement and hypothesised an aerobic metabolism [16, 31, 96]. In this study, we identified oxidoreductases, transferases and lyases (Additional file 4: Dataset S3) related to energy metabolism, gathering more evidence on *S. chrysophrii* metabolism. GO analyses revealed biological processes related to glycolysis, gluconeogenesis and the pentose-phosphate pathways (Fig. 4A), all indicative of an aerobic metabolism. Similar results were observed in other neodermates, including other Polyopistocotyla at a transcriptomic level [16], ESPs of Trematoda [79] and EVs from Trematoda and Cestoda [44, 97].

In addition to the previously discussed proteins, proposed EV-marker proteins such as 14–3-3 and heat shock protein 70 (HSP70), commonly found in eukaryotic EVs, were identified in *S. chrysophrii* vesicles (Additional file 4: Dataset S3), in agreement with other helminth species [54, 81, 86, 98]. Heat shock protein 90 (HSP90), previously detected in *S. japonicum* EVs [99], and four out of eight subunits of the T-complex protein 1 (TCP1) ring complex, a group II chaperonin related to heat shock protein 60 (HSP60) exhibiting ATP-dependent protein folding (Additional file 4: Dataset S3) [100–102], previously identified in *F. hepatica* [44], were also identified in *S. chrysophrii* EVs. The presence of HSP70/90 and TCP1 is reflected in the biological processes (Fig. 4A) and molecular functions (Fig. 4B) inferred from the GO analyses.

Other suggested EV-marker proteins include those with an EF-hand domain (PF13499), potentially characteristic of Trematoda and Cestoda EVs protein cargo [98]. In the *S. chrysophrii*-derived EVs in this study, three proteins presenting an EF-hand domain (PF13499.9; sorcin, calmodulin and calcineurin B homologous protein 1; Additional file 4: Dataset S3) were identified.

Currently, treatment options for polyopisthocotylidan infections in aquaculture, including *S. chrysophrii*, are limited, with hydrogen peroxide and formaldehyde baths in their licensed formulation being the sole available chemotherapeutants. However, formaldehyde baths have already been banned in Italy, among other countries, due to safety concerns, and its use will not be warranted worldwide in the coming years [5, 103, 104]. Coupled with the ongoing threat of anthelmintic drug resistance, these limitations underscore the need for further therapeutic or prophylactic strategies to address sparicotylosis and proactively combat emerging drug resistance. Most of the identified proteins in *S. chrysophrii* EVs have already been confirmed in other helminths including Neodermata [98]. Several proteins including the aforementioned peptidases, non-enzymatic proteins and energy metabolism-related proteins have been proposed as drug targets or vaccine candidates in Trematoda, Nematoda and certain Apicomplexa parasitic species. Table 4 compiles these protein target candidates from *S. chrysophrii* EVs, contributing to a comprehensive understanding of novel strategies against this parasite.

**Table 4:** List of orthologues described as drug and vaccine target candidates from different hematophagous parasites including Trematoda, Nematoda and Apicomplexa found in the EVs’ proteomic study using *Sparicotyle chrysophrii, Protopolystoma xenopodis*, *Gyrodactylus salaris* and *Microcotyle sebastis* proteomes as reference

Protein target candidates	References	Species and sequence hit
		***Sparicotyle chrysophrii***
*Proteases*		
Leucine aminopeptidase (M17 family)	[52, 73, 105–108]	maker-contig_20098_pilon_pilon-augustus-gene−0.16-mRNA-1
Alanyl aminopeptidase (M1 family)	[73, 109]	maker-contig_6883_pilon_pilon-snap-gene−0.23-mRNA-1
Calpain	[52]	maker-contig_24278_pilon_pilon-snap-gene−0.33-mRNA-1
*Carrier proteins*		
Ferritin	[84]	maker-contig_34520_pilon_pilon-augustus-gene−0.7-mRNA-1
*Cytoskeleton*		
Dynein light chain	[52, 107]	maker-contig_41206_pilon_pilon-augustus-gene−0.3-mRNA-1
14–3-3 protein	[52, 107]	maker-contig_34517_pilon_pilon-snap-gene−0.19-mRNA-1
		maker-contig_34517_pilon_pilon-snap-gene−0.19-mRNA-1
		maker-contig_30173_pilon_pilon-snap-gene−0.10-mRNA-1
Heat shock protein 70	[52, 107]	augustus_masked-contig_13257_pilon_pilon-processed-gene−0.1-mRNA-1
		augustus_masked-contig_5207_pilon_pilon-processed-gene−0.2-mRNA-1
Annexin	[52]	maker-contig_38531_pilon_pilon-exonerate_est2genome-gene−0.0-mRNA-1
		maker-contig_29652_pilon_pilon-augustus-gene−0.2-mRNA-1
		maker-contig_12610_pilon_pilon-snap-gene−0.5-mRNA-1
Tubulin	[110]	maker-contig_8332_pilon_pilon-augustus-gene−0.19-mRNA-1
		snap_masked-contig_2563_pilon_pilon-processed-gene−0.25-mRNA-1
*Antioxidant/ Defence/ Detoxification*		
Glutathione *S*-transferase	[52, 107]	maker-contig_7282_pilon_pilon-snap-gene−0.22-mRNA-1
Superoxide dismutase	[111]	maker-contig_45344_pilon_pilon-augustus-gene−0.2-mRNA-1
*Metabolism*		
Enolase	[52, 107]	snap_masked-contig_11270_pilon_pilon-processed-gene−0.1-mRNA-1
Fructose-bisphosphate aldolase	[107] [111]	maker-contig_31661_pilon_pilon-snap-gene−0.18-mRNA-1
		***Protopolystoma xenopodis***
*Cytoskeleton*		
Heat shock protein 70	[52, 107]	A0A448WN47
Tubulin	[110]	A0A448X3E2
		A0A3S5FFD8
		A0A448WX98
		A0A448XD95
		***Gyrodactylus salaris***
*Cytoskeleton*		
Tubulin	[110]	Q709M4
		***Microcotyle sebastis***
*Cytoskeleton*		
Annexin	[52]	B3GQS3

## Conclusions

In the current study, EVs have successfully been isolated and observed through TEM in the parasite’s clamp syncytial tegument of the haptoral region. Further protein characterisation from isolated *S. chrysophrii* EVs revealed a similar protein profile as in other neodermates, with several proteins related to EV biogenesis. The present findings allow the deepening of the understanding of aspects related to *S. chrysophrii* aerobic metabolism, iron transport, detoxification and proteolysis, among others, and overall identify therapeutic target candidates previously reported for other parasitic organisms.

### Supplementary Information

**Additional file 1: Dataset S1.** Spreadsheet containing information on the genomic in silico identification of proteins involved in multiple extracellular vesicle biogenesis pathways. Tab A has information on *Sparicotyle chrysophrii* genome and transcriptome dataset interrogation with *Homo sapiens* query sequences. Tab B has information about *Sparicotyle chrysophrii* genome dataset interrogation with query sequences belonging to diverse Neodermata species.

**Additional file 2: Dataset S2.** Spreadsheet containing information from the nanoparticle tracking analysis about the excretory/secretory products nanoparticle concentration according to size at each sampling point (24 h: tab A, 48 h: tab B and 72 h: tab C).

**Additional file 3: Figure. S1.** Nanoparticle concentration in the *Sparicotyle chrysophrii *in vitro maintenance media as a negative control (A) and 0.2 µm-filtered PBS as internal quality control (B). Shadowed areas correspond to the standard deviation.

**Additional file 4: Dataset S3.** Spreadsheet containing information on the proteome analysis of isolated extracellular vesicles from *Sparicotyle chrysophrii*. The spreadsheet contains sequences classified as non-enzyme proteins (tab A), unassigned Enzyme Commission number (tab B), oxidoreductase (tab C), transferases (tab D), hydrolases (tab E), lyases (tab F), isomerases (tab G), ligases (H) and translocases (tab I).

**Additional file 5: Dataset S4.** Spreadsheet containing information related to the ReviGO output. Tab A contains GO terms related to biological processes. Tab B contains GO terms related to cellular components. Tab C contains GO terms related to molecular functions.

## Data Availability

The proteomic data that support the findings of this study are openly available in Mass Spectrometry Interactive Virtual Environment (MassIVE) repository at http://massive.ucsd.edu/ProteoSAFe/status.jsp?task=35be3c4268c243ac8b0b2ccc63656244. Nucleotide and amino acid sequences from hypothetical proteins supporting the findings of this study are available upon request in ZENODO at https://doi.org/10.5281/zenodo.8405325. The rest of the data supporting the finding of this study are available in the supplementary material of this article.

## Abbreviations

BLASTp: Protein basic alignment search tool
BLASTx: Translated nucleotide sequence searched against protein sequences
BLOSUM: Block substitution matrix
ESCRT: Endosomal Sorting Complex Required for Transport
ESPs: Excretory-secretory products
EV: Extracellular vesicle
ILVs: Intraluminal vesicles
LC–MS/MS: Liquid chromatography with tandem mass spectrometry
MUSCLE: Multiple sequence comparison by log-expectation
MVBs: Multivesicular bodies
NTA: Nanoparticle tracking analysis
PASEF: Parallel accumulation/serial fragmentation
tBLASTn: Translated nucleotide basic alignment search tool
TEM: Transmission electron microscopy
