# Supplementary figures and images for: An inside out journey: biogenesis, ultrastructure and proteomic characterisation of the ectoparasitic flatworm Sparicotyle chrysophrii extracellular vesicles
Source: Parasit Vectors. 2024 Apr 3;17:175. doi: 10.1186/s13071-024-06257-x (PMC10993521; doi:10.1186/s13071-024-06257-x)

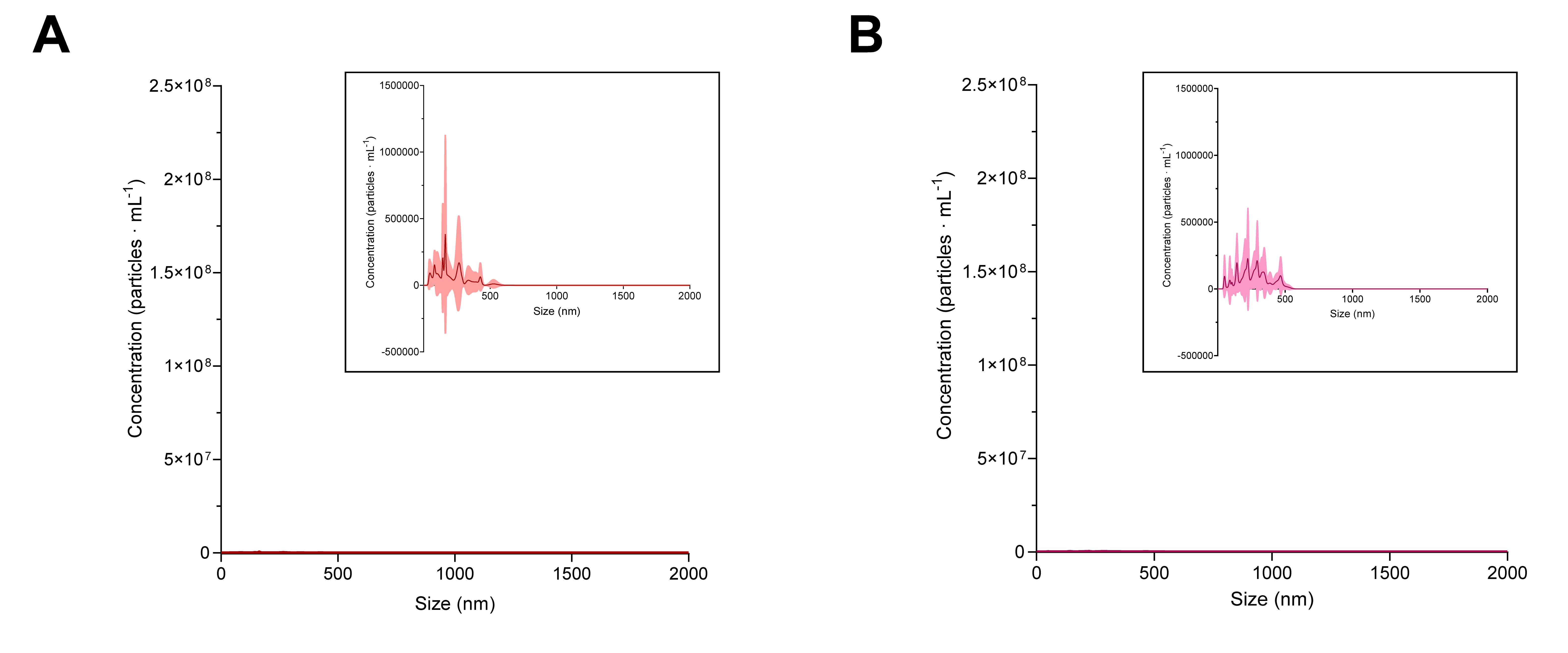

Supplement: Supplementary file 3 — Additional file 3: Figure. S1. Nanoparticle concentration in the Sparicotyle chrysophrii in vitro maintenance media as a negative control (A) and 0.2 µm-filtered PBS as internal quality control (B). Shadowed areas correspond to the standard deviation. [file 13071_2024_6257_MOESM3_ESM.tif]
